# The Aspirations of Measurement for Change

**DOI:** 10.3389/fpubh.2020.568677

**Published:** 2020-11-26

**Authors:** Joachim Krapels, Lotte van der Haar, Wiedaad Slemming, Joost de Laat, James Radner, Anselme Simeon Sanou, Penny Holding

**Affiliations:** ^1^Porticus Global, Amsterdam, Netherlands; ^2^Utrecht Centre for Global Challenges, Utrecht School of Economics, Utrecht University, Utrecht, Netherlands; ^3^Department of Paediatrics and Child Health, School of Clinical Medicine, University of the Witwatersrand, Johannesburg, South Africa; ^4^Munk School of Global Affairs & Public Policy, University of Toronto, Toronto, ON, Canada; ^5^Department of Public Health, Centre Muraz Biomedical Research Institute, Bobo Dioulasso, Burkina Faso; ^6^Saving Brains Collaborative Learning Team, Bromyard, United Kingdom

**Keywords:** measurement, evaluation, learning, context, stakeholder

## Abstract

This Perspective presents the five key aspirations of an approach to data use, decision making and monitoring, evaluation, and learning (MEL) in Early Childhood Development (ECD) referred to as Measurement for Change. The core ideas of Measurement for Change gave rise to this series, and many of the papers submitted in this series speak to this approach, whether directly or indirectly. The five aspirations describe interconnected concepts that advocate for practitioners and researchers within ECD to build the capacity to use data in their decision making, by establishing a monitoring, evaluation, and learning system that strives to be: Dynamic; Inclusive; Informative; Interactive; and People-centered.

## Introduction

The 2016 Lancet series *Early childhood development: the foundation of sustainable development* called attention to “estimates … that 250 million children (43%) younger than 5 years in low-income and middle-income countries are at risk of not reaching their developmental potential” [(1): p. 77]. The series highlighted the costs and risks of inaction, the value of interventions, and the need for government engagement to achieve better outcomes at scale (2). Yet, there is a considerable challenge to deliver high quality ECD interventions for all children, especially for those living in adversity; as a result, too few children in low resource settings are reached by high quality ECD programs (3, 4). Achieving success over whole populations, not just in small intervention settings, requires the combined knowledge, skills and expertise of multiple disciplines and professions, working at multiple levels, especially with the community and with families (5).

While the challenge remains to achieve widespread access to quality resources, the publication of the Lancet series in 2016, among others, underscores the growing global interest in ECD, and not only among academics and researchers. ECD practice similarly has in recent years seen an increase in investment and the development of national and international ECD policies (1). One key example is the United Nations' Sustainable Development Goal 4, target 4.2: “By 2030, ensure that all girls and boys have access to quality early childhood development, care, and pre-primary education so that they are ready for primary education” (6). Another is the launch of the Nurturing Care Framework, an initiative by WHO, UNICEF, World Bank, and others, focused specifically on translating this ambition on ECD into action (7).

There is growing recognition that the success of an ECD intervention at scale is not simply the function of an increase in size and reach (3–5, 8, 9). Scaling frequently diminishes or even eliminates the positive effects seen at small scale. With scale, the elements surrounding the delivery of an intervention frequently change. Even if the content or curriculum of an intervention remain the same, consistency in effectiveness cannot be assumed as key delivery mechanisms may need to alter. Adaptations may be required in light of new delivery agents (e.g., different teachers, health workers), different target groups (e.g., different children, parents), and differences in prevailing social and cultural norms and economic circumstances (10). Effective transfer of an intervention to the new setting may result in changes to content, implementation processes or both. Success is therefore the product of a range of components and processes, yet the aspect of local adaptation and implementation is understudied, and under reported, in comparison to the study of the effectiveness of individual intervention models.^1^ Two recent special issues speak to these challenges and, like the 2016 Lancet series, highlight the importance of strengthening monitoring and evaluation as a key contributor to generating knowledge on effective delivery at scale.

The 2018 special issue of *Annals of the New York Academy of Sciences*, “Implementation Research and Practice for Early Childhood Development,” harvested research findings on implementation of ECD programs. Contributors presented frameworks for gathering and reporting data relevant to effective implementation and called for continued empirical research with the explicit aim of improving both implementation and scale-up (3). The commentary paper to the series (12) builds on the contributions to provide seven recommendations providing “guidance on what implementers need to do in the coming years to provide a systematic body of useful knowledge …” [(12): p.265].

The 2019 *Archives of Disease in Childhood* series “Informing Design and Implementation for Early Child Development Programs” presented five papers and an editorial on ECD intervention design, measurement, and implementation topics. This series supports decision-makers in the design, implementation and program renewal cycle, with further calls for systematic data collection and reporting and, in common with the other two series, a focus on the challenge of successful scaling (9).

Each of the special issues or series (2016, 2018, and 2019) calls on implementers to continue strengthening their monitoring, evaluation and learning (MEL) systems to guide design, implementation and scale-up to create a systematic body of useful knowledge. However, there is also recognition of the challenges that arise when theory meets practice. For example, in one contribution the authors note that while interviewees^2^ agree on the importance of MEL, “data collection in ECD projects was often seen as intended solely for scientific publications, and of little use for project improvement or addressing implementation challenges” [(9): p. S47].

Given the importance of MEL highlighted by these special publications, this series intends to focus on the lived experiences of ECD implementers, harnessing the knowledge, know-how and practical solutions they are generating as they integrate data collection and MEL into their intervention design and implementation. As such this series hopes to stimulate the development of more effective, feasible MEL systems to support decision making at every stage of a program, from initial design through implementation and scaling, with ongoing adaptation. This implies engaging all those who work on ECD interventions, not just those who have been tasked with running MEL systems, in reviewing their systems, and the rigor with which they have tackled the need for ongoing adaptation. The ideas put forth in this series may also extend beyond the ECD sector, to others working on effective behavior change interventions in different fields.

The series will seek to explore how on-the-ground innovators are approaching questions such as: What does it mean for design and scaling decisions to be data and evidence informed? How can MEL contribute to decision-making that actively engages stakeholders, including beneficiaries? How can MEL adequately capture interactions between participants in the system, to better understand the sources of variability in outcome? How can MEL support the design of programs that move beyond the average, and become more responsive to specific or individualized needs? And, how can implementers manage the ethical dimensions of data collection and use? We refer to this approach as *Measurement* ***for****Change*, and distinguish it from *measurement of change*, that focuses more specifically on impact alone.

## Consultation and Co-Creation With ECD Practitioners

The aspirations of Measurement for Change emerged through consultation and co-creation, as described in detail in an accompanying Perspective (13). An inital approach was presented to ECD-practitioners, researchers and MEL specialists, mostly working in low-resource settings, for reflection and review. Through two workshops^3^, the first in Utrecht, the Netherlands in September 2019, and the second in Wardha, India in March 2020, participants were asked to reflect upon their own information and data use, and their MEL systems. Discussions brought into focus scaling challenges while identifying meaningful applications of monitoring, evaluation, and learning. The oral and written feedback provided by workshop participants on the initial set of aspirations was used to further develop the Measurement for Change approach. During the process of manuscript preparation by the Utrecht workshop participants developing papers for this series, we also drew upon their later reflections through a webinar in March 2020, and through an online survey in June 2020.

The purpose of this Perspective is to share with the reader the current framing of the Measurement for Change approach, and to provide a lens through which the reader can explore the value and feasiblity of the approach as described through the practical examples shared in the series. The series “*Effective Delivery of Integrated Interventions in Early Childhood: Innovations in Evidence Use, Monitoring, Evaluation and Learning”* includes narratives from Utrecht workshop participants and other contributors on innovations in monitoring, evaluation, learning, and data use.

## The Five Aspirations of Measurement for Change

Out of the consultation and co-creation process emerged five aspirations that currently inform the Measurement for Change approach. These five aspirations communicate an ambition for how MEL can function as ECD interventions are designed, implemented, and scaled. Measurement for Change addresses the connection between data collection and its utilization to help communities and families create nurturing, stimulating spaces where children can flourish.

The five aspirations describe interconnected concepts that together suggest that practitioners and researchers within ECD use data, decision making, monitoring, evaluation, and learning in a way that strives to be:

- **Dynamic**: with the capacity to adjust frameworks, processes or methods to be responsive to challenges, surprises, or opportunities, and to be able to reach learning goals
- **Inclusive**: with the capacity to identify and actively involve all stakeholders in making contributions to, and benefiting from, measurement and learning
- **Informative**: with the capacity to continuously seek, assess and use information from various sources to guide decision-making
- **Interactive**: with the capacity to observe, track and utilize interactions, responses, and relationships
- **People-centered**: with the capacity to be responsive to distinct and different goals, strengths, priorities, circumstances, characteristics of different people, and communities

As aspirations they serve to expand our thinking on why and how measurement, in its various ways and forms, can be utilized to create effective programs serving families and children. Cross-cutting these five aspirations is a focus on human dignity and a human rights centered approach. At the heart of the Measurement for Change concept lies a recognition of the dignity of every person, including, notably, the child and her family members. An ECD system must honor and respect that dignity in every relationship engaged in, and in every activity undertaken, including those focused on measurement, monitoring, evaluation, and learning. Through respectful, collaborative engagement, systems serving families and children will generate valuable learning and evidence-based insights.

### Dynamic

A “dynamic” approach embeds an ability to adjust and adapt frameworks, processes, or methods in response to changing circumstances. An implementation process will face challenges, unexpected or otherwise, new opportunities, surprises, new information and lessons, that will influence the achievement of the targeted impact or quality of delivery. A dynamic approach anticipates that as projects or interventions progress, knowledge and information will increase, which will inform new adaptations. While the end goals may remain constant, a dynamic approach leaves flexibility in how those goals can be achieved and seeks to base adjustments on information gathered. A dynamic approach implies an intentional awareness of the context, stakeholders, and of future plans, such as scaling. This awareness resonates strongly with the other aspirations, which highlight different ways in which we can intentionally be sensitive in our measurement.

A paper submitted to this Series from a team in Bulgaria provides an illustration of a dynamic approach to MEL. A randomized controlled trial was insufficient to achieve the aim of informing change in national ECD policy. Additional evidence answering complementary questions was needed, ranging from municipal level data and case studies for assessing local situations, to a nationally representative survey of parental attitudes toward the proposed policy change, among others.^4^ The reader is invited to explore articles in the series for further illustrations on how this aspiration can be achieved while also maintaining a systematic and rigorous methodology which tracks and explains pathways to change.

### Inclusive

The aspiration to be inclusive captures the intention and the capacity to include all stakeholders, the children, families and communities, in the development and use of measurement within interventions. This aspiration is linked to the principle that distributed leadership, constructed around a common goal, is a cornerstone of the human rights approach. We also hypothesize that the generation of a system of distributed leadership will be an asset for scaling, as interventions adapt to different contexts and cultures.

The Inclusive aspiration addresses the crucial role that all stakeholders, from the children to the policy makers, play in the development, implementation and sustainability of an intervention, and the key role they have in defining and assuring success. The diversity of contributions to the conversations at any stage of program development will increase the value of the learning and insights that can be derived from measurement. As elaborated under the Informative aspiration below, key stakeholders, including the children, families and communities, are a crucial source of knowledge, which needs to be engaged for interventions to be adopted and owned at a local level. Being inclusive means that stakeholders should be included not simply as sounding boards, or occasional contributors, but as genuine partners in a joint project. For example, the paper in this series by Nair et al. (14) describes efforts to improve inclusion of fathers, a crucial stakeholder group, in an ECD intervention in India.

The ambition to be inclusive can seem overwhelming, as it may appear everything and everybody will need to be included at all times. Resource and other constraints will inevitably balance the extent to which stakeholders can reasonably and practically be included. The aspiration nevertheless urges us to look at measurement from the perspective of *inclusion*; that is Measurement for Change seeks, from the outset and always, to recognize and respect the human rights of stakeholders and to include them intentionally. Clear documentation of how and to what extent inclusion is achieved, as well as the rationale for limitations, will contribute valuable information to guide future planning.

### Informative

“Informative” is the ambition for the measurement system to have the capacity to continuously inform decisions by bringing to bear a wide variety of relevant sources of information, at all stages of project development and implementation. As such this aspiration links to the Dynamic aspiration, given the evolving nature of information needed to inform decisions, and to the Inclusive aspiration, since multiple perspectives are relevant to good decision-making. Thus, the Informative aspiration signals a commitment to learning from the context and from all stakeholders. Respect for those stakeholders suggests that no individual can a priori know what is the best solution for a particular problem or situation; rather, solutions will emerge from a dialogue that combines local insight with global knowledge.

Information is understood in a broad sense, based on diverse kinds of data that may be generated, analyzed and used to support decision-making, and the different forms of dialogue and engagement that the Inclusive aspiration encourages. If it is true that successful scaling relies on a variety of skills, expertise, experiences, and knowledge, as Shonkoff et al. (5) note, then the sources of information and knowledge to be generated and drawn upon ought to be equally varied. Likewise, use of information should not be limited to applying conclusions from formal processes or products, such as research studies or evaluations. Rather, feedback and other relevant information should be continuously generated and used.

This process was followed in an early intervention program for children with delayed development in rural areas of India. The program relied on rapid evaluation cycles to improve design and delivery, informed by mixed methods such as surveys, focus groups and interviews.^5^ The richness of the data, and rapid data collection and analysis cycles, allowed for improvements to the program on an almost continuous basis to prepare it for scaling. Readers are invited to reflect on the diversity and utility of their own data sources as they examine the details of the different examples described in the series.

### Interactive

The next aspiration recognizes the complexity of ECD, where the pathway to change is seldom linear. The Interactive aspiration captures the capacity to measure the cyclical nature of interactions and responses between and among adults and children that is inherent in social behavior. This contrasts with a linear model of change, where influence flows from one stakeholder to another, for example from a parent to a child. Failure to explore and respond to the interactive “feedback loop” may lead to cessation of a potentially valuable activity and can, at its most extreme, negatively impact development and change. The Interactive aspiration implies the capacity to observe, track and utilize interactions, responses, and relationships.

For example, at the level of a single parent child dyad, if a baby cries and the caregiver fails to respond (the baby is left to cry), initially the cries will increase. Should the caregiver continue with no response, eventually the crying will cease altogether. In the most extreme of these situations the child will develop an attachment disorder negatively impacting their socio-emotional development, including their ability to form healthy relationships in the future. In another example, when a caregiver coos at the baby and the baby fails to coo back, at first the caregiver is likely to coo more. With a continued lack of response, caregiver initiated cooing may cease altogether, negatively influencing that child's communicative development. These examples suggest that the exploration of the interaction, rather than just the initial response, should uncover the sources of the variability in responses, and lead to more effective interventions that have a sustainable benefit over time.

Many interactions include more than two participants, illustrated in the following example given by Cartwright and Hardie (10). In a parenting program focused on improving child growth the pathway of change was initially conceived as the transfer of knowledge to the mother, who will improve nutritional practices at home. After successful implementation in Tamil Nadu, the application of a similar program in Bangladesh failed to achieve the anticipated growth in children. An exploration of interactions beyond the direct participants, uncovered that it was family members *not* present at the sessions—for example, the mothers-in-law—who were important decision makers in the home. This knowledge led to the conclusion that including these other actors in the parenting sessions may be a more effective strategy than increasing the number of sessions. Including a method for exploring social interactions in the monitoring and evaluation process is crucial to understanding the underlying complexities that influence change.

### People-Centered

This aspiration brings to the fore natural diversity among people. No individual quite matches the average, no context wholly mirrors another. Central to the aspiration is that programs build the capacity to be responsive to the distinct goals, strengths, priorities, circumstances, and characteristics of different people and communities. It is commonplace that different people react differently to interventions. By exploring natural diversity, by measuring and analyzing beyond the average, MEL systems can contribute more effectively to a deeper understanding of the possibilities and potentials of human development. This is well-illustrated by a paper submitted to this Series on an intervention in the Amazon.^6^ The intervention involves 27 ethnic groups speaking 22 different languages. It honors this diversity by using digital media to record the different childrearing practices so they can be shared with, and are accessible to, others.

## Aspirations and the Series

The five core ideas that constitute Measurement for Change have been explicitly framed as *aspirations* as they reflect an approach to monitoring, evaluation, and learning that may be developed, in different ways, in the context of different interventions. Measurement for Change is not intended as a prescriptive framework, but rather as a guide to building the capacity to choose the right method for the context, resources, and stage of development of an intervention or program.

In developing the Aspirations through the practical application of the practitioners we have worked with, we have seen their value as reference points for reflecting on all the stages of the development of an intervention as well as their usefulness as guides for building a path to scale. As such, the Aspirations can be seen as complementary to existing implementation science frameworks.^7^By applying the Aspirations across the elements of alternative frameworks, a practitioner can enhance and deepen the value of the method they choose to use.

We hope the monitoring, evaluation, and learning efforts of ECD practitioners discussed in the articles in this series will shed light on whether and how the aspirations proposed provide guidance, and how they are being shaped in practice. The articles explore the efforts taken to apply them, and what the processes followed may ultimately mean for the well-being of children and families participating in ECD interventions.

Collectively, these contributions may validate the framing of the aspirations, or raise areas for improvement and adaptation. The exciting prospect for this collection of papers is to explore how this series can give both practical content to the aspirations, while also contributing to the further development of a monitoring, evaluation and learning system that uses measurement to promote and enhance change, rather than merely record the change that has already occurred.

## Data Availability Statement

The original contributions presented in the study are included in the article/supplementary material, further inquiries can be directed to the corresponding author.

## Author Contributions

JK led on writing and editing, but substantial written contributions were provided by LH, WS, JL, JR, AS, and PH. All authors contributed equally to the development of the ideas presented in this Perspective.

## Conflict of Interest

The authors declare that the research was conducted in the absence of any commercial or financial relationships that could be construed as a potential conflict of interest.

## Footnotes

^1^Cartwright (11) contrasts the “intervention-centered approach” with to a “context-centered” approach, and argues that while both are valuable “we need to put far more effort” (p. 54) into the latter; see also Radner et al. (4).

^2^32 interviewees: 13 were from NGOs, 17 from academic institutions, 1 independent consultant from the private sector, and 1 policymaker.

^3^Attendees in the workshop at Utrecht University, in Utrecht, represented 21 organizations in ECD from across the globe. Attendees of the workshop at the Datta Meghe Institute for Medical Sciences, in Wardha, represented 11 organizations working in the ECD-field in India.

^4^Paper submitted to this Series by Volen and De Laat titled “Building Evidence for Preschool Policy Change in Bulgaria.”

^5^Paper submitted to this Series by Krishna et al. titled “Rapid-cycle evaluation in an early intervention program for children in South India: optimizing service providers' quality of work-life, family program engagement and school enrollment.”

^6^Paper submitted to this Series by Shaw and De Cácia Oenning da Silva titled “Look Upstream: Measurement for Innovation on the Upper Rio Negro of the Amazon Basin.”

^7^An example of such a framework would be the Medical Research Council's Guidance on developing and evaluating complex interventions (15). But equally the Aspirations seem to complement, as another example, the toolkit for engagement of communities described by Pratt (16). In addition, a simple four step model was used in the workshops with practitioners on Measurement for Change to stimulate the exchange of ideas, as described by Van der Haar et al. (13).
